# A Comparative Analysis of Pre-Exposure Prophylaxis Awareness, Acceptance, and Barriers Among Populations of Men Who Have Sex with Men in Global Settings: An Integrative Literature Review

**DOI:** 10.3390/nursrep16050148

**Published:** 2026-04-22

**Authors:** Won Ju Hwang, Hwiyun Kim, Nancy R. Reynolds

**Affiliations:** 1 College of Nursing Science, Kyung Hee University, Seoul 02447, Republic of Korea; kimhy1509@khu.ac.kr; 2 School of Nursing, Johns Hopkins University, Baltimore, MD 21205, USA; nancy.reynolds@jhu.edu

**Keywords:** pre-exposure prophylaxis (PrEP), men who have sex with men (MSM), integrative literature review, awareness–uptake gap, nursing

## Abstract

**Background**: Although pre-exposure prophylaxis (PrEP) has demonstrated strong clinical efficacy in preventing HIV infection among men who have sex with men (MSM), real-world utilization remains suboptimal. In South Korea, MSM constitute a major population within the domestic HIV epidemic; however, PrEP uptake has not increased pro-portionally to awareness. This discrepancy has been conceptualized as the “awareness–uptake gap,” reflecting multi-level barriers beyond individual knowledge. **Purpose**: This integrative review aimed to compare PrEP awareness, acceptance, and utilization among MSM populations in South Korea and international settings, and to identify structural, institutional, and psychosocial determinants contributing to the awaness, uptake gap. The study further sought to derive practical implications for nursing practice and health policy. **Methods**: An integrative literature review was conducted following Whittemore and Knafl’s five-step methodology and reported in line with PRISMA guidance. Electronic searches were performed in PubMed, Google Scholar, RISS, ScienceON, and DBpia for peer-reviewed studies published between 2015 and 2025 in English or Korean. The final search was completed on 31 January 2026. A total of 5952 records were identified, and 187 studies met the inclusion criteria after screening and duplicate removal. Quality appraisal was conducted using AXIS, Newcastle-Ottawa Scale, RoB 2.0, CASP, and MMAT according to study design, and the findings were synthesized within an environmental–structural–individual framework. **Results**: The included studies consistently showed that awareness of PrEP exceeded actual uptake. Across settings, the awareness–uptake gap was shaped by policy environment, service accessibility, stigma, privacy concerns, economic burden, institutional complexity, and provider preparedness. Comparative evidence from China, Thailand, Belgium and France, Brazil, and West Africa further suggested that awareness alone did not ensure uptake when service pathways were fragmented, culturally unsafe, or poorly understood. **Conclusions**: Closing the awareness–uptake gap requires integrated policy and practice strategies that extend beyond cost reduction. Strengthening confidentiality systems, simplifying service pathways, and enhancing provider competency—particularly through nurse-centered PrEP navigation and counseling models—may support more sustainable PrEP expansion among MSM populations in global settings.

## 1. Introduction

### 1.1. Rationale for the Study

Human Immunodeficiency Virus (HIV) infection outcomes have improved dramatically with advances in antiretroviral therapy (ART). Nevertheless, HIV risk remains disproportionately concentrated in certain populations. In particular, men who have sex with men (MSM) have consistently been identified as a key population at elevated risk of HIV infection compared with the general population across diverse regions and epidemiological contexts [1]. The South Korean context warrants particular attention, given the substantial proportion of transmission associated with MSM. According to the Korea Disease Control and Prevention Agency (KDCA) 2024 HIV/AIDS Annual Report on Reported Cases, 975 newly reported HIV infections occurred in 2024. By age group, individuals in their 30s accounted for 36.9% (360 cases), followed by those in their 20s at 29.8 (291 cases), and those in their 40s at 13.7% (134 cases), indicating that 66.7% of new infections occurred among adults aged 20–40 years. Additional distributions were reported as follows: individuals in their 50s, 10.5% (102 cases); 60s, 5.6% (55 cases); teens, 1.3% (13 cases); and 70 years or older, 2.1% (20 cases). Among cases responding to epidemiological investigations regarding route of infection, sexual contact accounted for the vast majority (99.8%), and among these, same-sex sexual contact comprised the largest share (63.7%), demonstrating that MSM constitute a major population group within the domestic HIV epidemic [2].

Within this context, HIV prevention strategies have increasingly emphasized biomedical prevention, including pre-exposure prophylaxis (PrEP) as a key preventive modality [3,4]. A representative strategy is PrEP, in which HIV-negative individuals take antiretroviral medication preventively to reduce infection risk. Key clinical studies such as iPrEx support PrEP as a powerful preventive intervention for MSM populations [3]. However, demonstrated preventive efficacy does not automatically translate into real-world adoption and scale-up. South Korean studies have reported that even when PrEP is perceived as necessary, limitations in insurance coverage and insufficient knowledge function as major barriers to access [5]. More recently, a domestic PrEP pilot and demonstration initiative highlighted retention and counseling systems as key issues during real-world service delivery [6].

Barriers to PrEP dissemination cannot be reduced solely to individual knowledge or attitudes. Social stigma related to PrEP use and concerns about privacy disclosure may structurally constrain PrEP access and sustained use, thereby creating gaps between the stages of awareness, acceptance, and actual use. Accordingly, this study uses an integrative literature review approach to comparatively synthesize domestic and international literature on PrEP among MSM populations and to organize determinants and barriers operating across the stages of awareness, acceptance, uptake, and continuation. By deriving practical strategies and policy implications that healthcare practitioners, including nurses, can implement in community and clinical settings, this study aims to build an evidence base to improve PrEP accessibility among MSM populations.

### 1.2. Study Objective

The objective of this study is to compare and analyze PrEP awareness, acceptance, and actual utilization among MSM populations in South Korea and international settings and to identify determinants and structural barriers that generate the awareness–uptake gap between awareness and actual utilization. Through this analysis, the study seeks to explore practice strategies in community health and healthcare settings to improve PrEP accessibility for MSM in South Korea and to provide practical implications for establishing evidence-based counseling and management systems that can be implemented by practitioners, including nurses. In this review, awareness refers to knowledge of the existence of PrEP; acceptance refers to a favorable attitude or willingness to use PrEP; and uptake refers to actual initiation or use of PrEP.

## 2. Methods

### 2.1. Study Design

This study was conducted as an integrative literature review, consistent with Whittemore and Knafl’s methodology [7], to synthesize empirical evidence on PrEP awareness, acceptance, and uptake among MSM populations across diverse policy and institutional contexts. The review presented in the manuscript is an integrative review, and the methodological framing is standardized accordingly to avoid ambiguity. Preferred Reporting Items for Systematic reviews and Meta-Analyses (PRISMA) principles were used to increase transparency in study identification, screening, eligibility assessment, and reporting.

To organize the synthesis, an environmental–structural–individual framework was applied. In this framework, environmental factors refer to macro-level policy, legal, and financing conditions; structural factors refer to service delivery pathways, institutional accessibility, and confidentiality procedures; and individual factors refer to knowledge, attitudes, perceived stigma, and willingness to use PrEP. This framework guided the coding, grouping, and interpretation of the included studies rather than serving only as a descriptive label.

### 2.2. Study Procedure

This integrative review followed the five-step process proposed by Whittemore and Knafl [7]: problem identification, literature search, data evaluation, data analysis, and data presentation. In the problem identification stage, the core issue was defined as the awareness-uptake gap, that is, the repeated observation that awareness of PrEP among MSM populations does not automatically translate into actual uptake. In the literature search stage, domestic and international databases were searched systematically to identify studies published between 2015 and 2025. Screening and data handling procedures were prespecified, and the same eligibility criteria and extraction matrix were applied by two reviewers throughout the review process.

In the data evaluation stage, records were screened against predefined eligibility criteria at the title and abstract level and then reassessed through full-text review. In the data analysis stage, the included studies were organized into a standardized extraction matrix that captured study year, country or region, study design, sample characteristics, recruitment strategy, whether MSM-specific findings were reported separately, key outcomes related to awareness, acceptance, uptake, or continuation, and the main policy, structural, psychosocial, and provider-related findings. Data extraction was completed independently by two reviewers using the same matrix as a form of double data extraction, and discrepancies were reconciled through discussion.

Because the included literature was heterogeneous in design, measurement, and reporting, a statistical meta-analysis was not attempted. Instead, the findings were narratively integrated within the environmental–structural–individual framework, with attention to shared patterns across regions, region-specific differences, and implications for nursing practice.

### 2.3. Problem Identification

This study defines the core problem as the awareness–uptake gap, wherein increasing PrEP awareness among MSM populations has not been matched by corresponding increases in actual utilization. Despite expanded dissemination of PrEP information and efforts to improve accessibility, repeated drop-off at the utilization stage cannot be sufficiently explained by simple information deficits or individual willingness alone. Rather, the lack of automatic conversion from awareness to uptake suggests a multi-layered outcome driven by interactions among healthcare systems, institutional arrangements, and social–structural conditions.

MSM populations may be exposed to psychosocial barriers such as stigma related to sexual minority status and HIV, experiences of discrimination in healthcare settings, and concerns regarding disclosure of personal information. In addition, structural factors such as rigidity of PrEP delivery systems, financial burden, fragmented service pathways, and limited gender- and sexuality-sensitive communication among healthcare providers may act as barriers that limit utilization regardless of individual prevention intent. Accordingly, this study does not treat the PrEP awareness–uptake gap as merely an issue of individual choice, but examines it within an integrated context shaped by interacting individual, environmental, and healthcare-system factors.

### 2.4. Literature Search

Studies addressing the gap between PrEP awareness and actual utilization among MSM populations were searched in PubMed (National Library of Medicine, Bethesda, MD, USA), Google Scholar (Google, Mountain View, CA, USA), RISS (Korea Education and Research Information Service, Daegu, Republic of Korea), ScienceON (Korea Institute of Science and Technology Information, Daejeon, Republic of Korea, and DBpia (Nurimedia, Seoul, Republic of Korea). Eligible publications were limited to peer-reviewed journal articles published between 2015 and 2025 in English or Korean.

The database-specific search strategy combined controlled vocabulary and free-text keywords related to population, intervention, and outcomes. Core concepts included “men who have sex with men” OR “MSM,” “pre-exposure prophylaxis” OR “PrEP,” and outcome or barrier terms such as “awareness,” “acceptance,” “willingness,” “uptake,” “utilization,” “access,” “barriers,” and “structural factors.” A representative PubMed strategy was: ((“men who have sex with men”[Title/Abstract] OR MSM[Title/Abstract]) AND (“pre-exposure prophylaxis”[Title/Abstract] OR PrEP[Title/Abstract]) AND (awareness[Title/Abstract] OR acceptance[Title/Abstract] OR willingness[Title/Abstract] OR uptake[Title/Abstract] OR utilization[Title/Abstract] OR access[Title/Abstract] OR barrier*[Title/Abstract] OR structural[Title/Abstract])). Equivalent keyword combinations were adapted for Google Scholar, RISS, ScienceON, and DBpia according to each platform’s search interface.

Because Google Scholar can yield very broad and low-specificity results, its operationalization was restricted to improve reproducibility. Searches were conducted using major concept blocks corresponding to MSM, PrEP, and awareness/uptake/barrier terms; results were sorted by relevance, and the first 10 result pages were screened for each major query combination. Duplicates and non-peer-reviewed materials were removed during screening.

Studies were included if they (1) focused primarily on MSM populations or reported MSM-specific findings separately within mixed key-population samples, (2) examined PrEP awareness, acceptance, willingness, uptake, access, continuation, or related barriers and facilitators, and (3) presented original empirical findings using quantitative, qualitative, or mixed-methods designs. Multinational or multicenter studies were retained when MSM-specific findings relevant to the review question were available. When overlapping samples were suspected, the most comprehensive report was retained, and additional reports were used only if they contributed distinct outcomes, follow-up periods, or contextual findings. To preserve the breadth expected in an integrative review while reducing the influence of weak evidence, studies assessed as low quality were excluded; studies rated as moderate quality were retained only when they met essential methodological criteria and contributed distinct contextual, implementation, or qualitative insights relevant to the review question.

The electronic database search yielded 2456 records from PubMed, 3060 from Google Scholar, 142 from RISS, 108 from ScienceON, and 186 from DBpia, for a total of 5952 records. Manual review of reference lists did not identify additional eligible studies. Two reviewers independently screened titles and abstracts, followed by full-text assessment of potentially eligible reports. Disagreements were infrequent and were resolved through discussion; formal inter-reviewer agreement statistics were not calculated.

### 2.5. Quality Appraisal

Quality appraisal was conducted for all 187 included studies according to study design. AXIS (The Critical Appraisal Guidelines for Cross-Sectional Studies, BMJ Open) was used for cross-sectional studies, the Newcastle-Ottawa Scale (Wells et al., Ottawa Hospital Research Institute, Ottawa, ON, Canada) for cohort and case–control studies, RoB 2.0 (Cochrane, London, UK)for randomized controlled trials, CASP (Critical Appraisal Skills Programme, Oxford, UK) for qualitative studies, and MMAT (v2018; McGill University, Montreal, QC, Canada) for mixed-methods studies. Two reviewers independently conducted the appraisal, and discrepancies were resolved through discussion or consultation with a third reviewer when needed.

For transparency, 120 studies were appraised with AXIS, 16 with the Newcastle-Ottawa Scale, 6 with RoB 2.0, 35 with CASP, and 10 with MMAT. Studies were classified as high quality when they met most core criteria within the relevant tool (e.g., AXIS ≥ 15/20 Newcastle-Ottawa Scale ≥ 7 stars, CASP positive judgment across the major domains, MMAT ≥ 4/5, or low overall risk in RoB 2.0). Studies that satisfied essential methodological criteria but did not meet the high-quality threshold were categorized as moderate quality. These studies were retained because integrative reviews aim to synthesize heterogeneous evidence, including contextual and implementation findings, while still excluding reports with major methodological limitations. Overall, 139 studies (74.3%) were classified as high quality and 48 (25.7%) as moderate quality.

A summary of the appraisal tools, applicable study designs, and quality classification thresholds is presented in Table 1.

### 2.6. Data Extraction and Synthesis

After eligibility assessment and quality appraisal, the included studies were reviewed repeatedly and charted in a standardized matrix to preserve the meaning and context of the original reports. The extraction matrix included study year, country or region, study design, sample characteristics, whether the sample was exclusively MSM or drawn from mixed key populations, awareness/acceptance/uptake-related outcomes, and the main environmental, structural, psychosocial, and provider-related findings. For descriptive purposes, studies were additionally classified at the report level as MSM-only samples or mixed key-population studies reporting MSM-specific findings separately.

**Figure 1:**
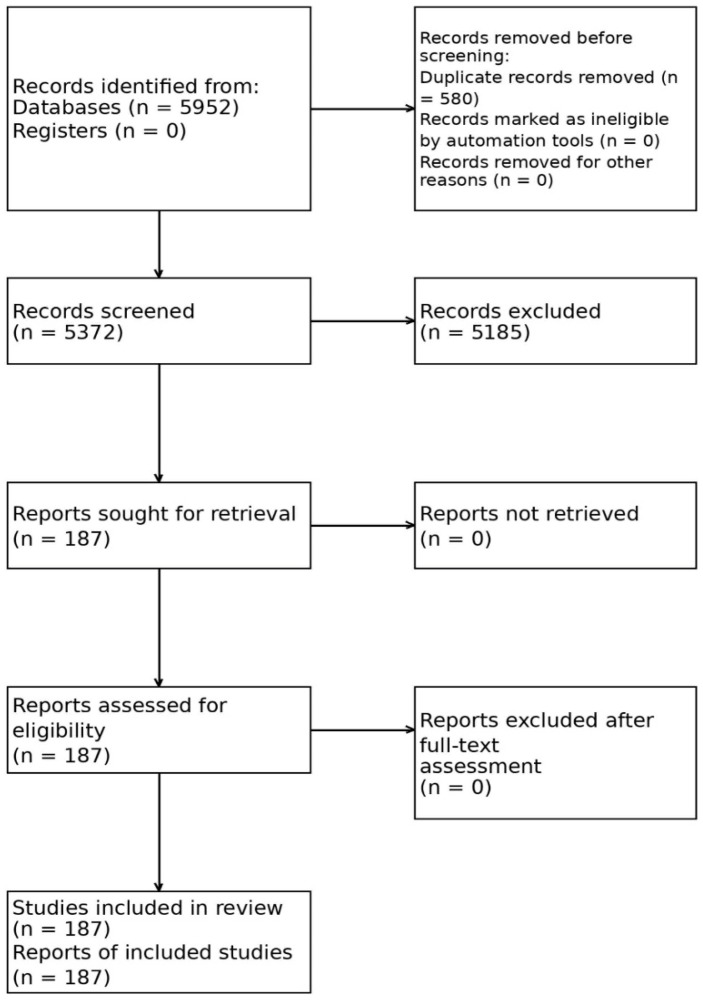
PRISMA flow diagram of study selection.

Findings were then synthesized thematically within the environmental–structural–individual framework. Environmental themes addressed policy, reimbursement, and public-health infrastructure. Structural themes addressed service organization, confidentiality, access pathways, and provider practices. Individual themes addressed awareness, willingness, perceived stigma, and behavioral uptake. During synthesis, the review distinguished between (1) findings that recurred across multiple settings, (2) barriers that appeared particularly salient in South Korea, and (3) issues that emerged more strongly in qualitative than in quantitative studies. This analytic approach was used to move beyond simple description and to support cross-context comparison (Figure 1 and Table 2).

## 3. Results

### 3.1. Characteristics of Included Studies

Among the 187 included studies, quantitative studies predominated (n = 141, 75.4%), followed by qualitative studies (n = 35, 18.7%) and mixed-methods studies (n = 11, 5.9%). Cross-sectional designs represented the largest share of quantitative work, whereas qualitative and mixed-methods studies contributed depth by illuminating mechanisms such as stigma, privacy concerns, and ambivalence toward PrEP use that are not easily captured through numerical indicators alone. Study-level classification further indicated that 156 studies (83.4%) used MSM-only samples, whereas 31 studies (16.6%) reported MSM-specific findings within broader key-population samples.

By publication year, 67 studies (35.8%) were published between 2015 and 2019 and 120 (64.2%) between 2020 and 2025, indicating increased research activity in the most recent period. Regarding regional distribution, North America accounted for 87 studies (46.5%), Asia for 35 studies (18.7%), and Europe and other regions for 65 studies (34.8%). The United States accounted for the single largest national share, followed by several Asian settings, including Thailand, China, and South Korea, suggesting growing international interest in PrEP implementation among MSM populations.

Among quantitative studies, samples of 100–499 participants were most common (n = 64, 45.4%), followed by 500–999 (n = 32, 22.7%), fewer than 100 (n = 25, 17.7%), and 1000 or more (n = 20, 14.2%). Quality appraisal showed that 139 studies (74.3%) were rated high quality and 48 (25.7%) moderate quality. Although methodological heterogeneity remained substantial, the included literature provided a broad evidence base for examining the awareness–uptake gap.

### 3.2. Institutional Foundations and Economic Accessibility of PrEP: How Policy Environments Shape Uptake

Across the included literature, the relationship between awareness and uptake was shaped not only by individual willingness but also by the extent to which PrEP had been institutionalized as a routine preventive service. A recurring pattern was that uptake was stronger when reimbursement, prescribing pathways, laboratory monitoring, and counseling systems were aligned, whereas fragmented access pathways appeared to widen the awareness–uptake gap.

In the United States, PrEP has increasingly been embedded within a preventive-service framework. The USPSTF Grade A recommendation, Affordable Care Act coverage mechanisms, the Ending the HIV Epidemic strategy, and CDC clinical guidance together created a comparatively coherent pathway linking eligibility assessment, prescription, monitoring, and follow-up [8,9,10,11,12,13]. This institutional coherence appears to reduce some economic barriers, although stigma, privacy concerns, and provider-level barriers remain.

In South Korea, by contrast, PrEP access appears more fragmented. Existing studies suggest that conditional reimbursement, public support programs, uncertainty regarding service pathways, and limited access to accurate information may hinder transition from interest in PrEP to sustained use [5,6]. This pattern suggests that financial support alone is insufficient when users still face uncertainty about where to seek care, how follow-up is organized, and whether confidentiality will be protected.

The broader international evidence showed that policy and access conditions varied across regions. In other Asian settings, including Thailand and China, studies described a mixture of expanding policy visibility and continued dependence on limited delivery sites, community organizations, or pilot-oriented pathways, with procedural complexity still constraining uptake [14,15,16,17]. In European settings such as Belgium and France, PrEP was more often embedded within reimbursed or clinically routinized prevention systems, yet uneven geographic access and regimen literacy remained important considerations [18,19]. In Brazil, formal public-sector expansion improved the visibility of PrEP but did not eliminate social inequity, uneven service concentration, or stigma [20,21,22]. Evidence from West and sub-Saharan Africa likewise suggested that willingness could be high once PrEP was explained, but awareness, affordability, and continuity of access remained inconsistent [23,24,25,26,27].

Taken together, the reviewed evidence suggests that policy alignment, clarity of service pathways, and confidentiality safeguards are central determinants of whether awareness can realistically translate into uptake. For South Korea, the comparative implication is not merely that cost should be reduced, but that access should become more navigable, standardized, and trustworthy within routine clinical and community health systems.

### 3.3. PrEP Awareness and Acceptance Among MSM Populations: Information Asymmetry and the Formation of the Awareness–Uptake Gap

Across the included studies, awareness of the existence of PrEP was generally more common than actual uptake, indicating that knowledge of PrEP alone was insufficient to produce sustained use. Acceptance appeared to depend not only on basic awareness, but also on whether potential users understood how PrEP works, how it is prescribed, how safety monitoring is conducted, and how much the care pathway would cost.

In the United States, PrEP awareness expanded after major efficacy data became widely disseminated and formal policy support strengthened the legitimacy of PrEP as a preventive service [3,8,9]. Even so, the literature indicates that high awareness did not consistently lead to uptake when stigma, perceived complexity, mistrust, or fear of disclosure remained unresolved [28,29,30].

In South Korea, awareness among MSM has improved, but the literature still suggests a gap between surface-level familiarity with PrEP and deeper procedural understanding. Korean MSM may encounter information through online communities and social media, yet uncertainty about side effects, long-term use, monitoring procedures, and costs may weaken acceptance and delay entry into care [5]. In this sense, information asymmetry operates not only between healthcare providers and users, but also between basic exposure to PrEP and the practical knowledge needed to initiate use safely.

More explicit regional comparison also showed that the awareness–uptake gap was not unique to South Korea. Thai and Chinese studies frequently described relatively high awareness or willingness accompanied by a marked drop-off at the level of initiation, adherence, or routine continuation, particularly when youth-friendly, community-linked, or clearly navigable services were lacking [14,15,16,17]. European studies suggested that even in more institutionalized settings, uptake still depended on dosing literacy, perceived candidacy for PrEP, and the visibility of service entry points [18,19]. In Brazil, awareness and willingness also coexisted with intersectional barriers such as internalized homonegativity, misinformation, and racialized or socially stratified access to services [21,22].

Overall, the available evidence supports an interpretation of the awareness–uptake gap as an informational and relational problem as much as a financial one. Awareness becomes actionable only when users can trust the service pathway, understand how care is delivered, and anticipate respectful, confidential interaction with providers.

**Figure 2:**
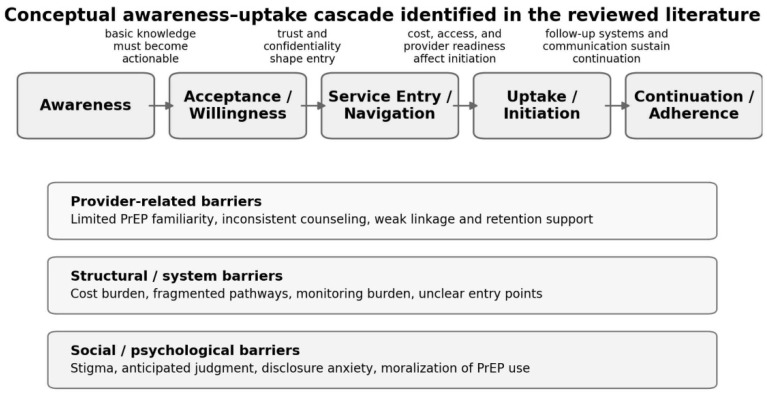
Conceptual awareness-uptake cascade and barrier domains identified in the review.

The cross-regional comparative synthesis is summarized in Table 3, and Figure 2 conceptually depicts how barrier domains accumulate across the awareness-uptake cascade.

### 3.4. Multidimensional Barriers Driving the Awareness–Uptake Gap: Stigma, Privacy, Institutional/System Factors, and Provider Factors

The reviewed literature indicates that the awareness–uptake gap is produced by interacting barriers rather than a single isolated factor. These barriers can be grouped into social and psychological barriers, institutional and system barriers, and provider-related barriers; however, the relative salience of each barrier likely varies by region and service context.

At the social and psychological level, stigma was a recurring theme. Studies described how PrEP use could be moralized or associated with sexual irresponsibility, creating hesitation even among individuals who understood the preventive value of PrEP [28,29,30]. Such stigma may operate through anticipated judgment, self-censorship, and avoidance of clinical settings. In the Korean context, stigma may also intersect with concerns about disclosure of sexual orientation and HIV-related assumptions, thereby intensifying reluctance to seek services.

At the institutional level, privacy concerns and fragmented access pathways raised the threshold for service entry. Anxiety that sexual behavior or sexual orientation might be disclosed, uncertainty about where to obtain PrEP, and the burden of repeated monitoring may all discourage service initiation or continuation [5,6,31]. Institutional complexity, therefore, appears to matter not only at the point of initial uptake but also across the continuity of PrEP care.

Global synthesis also clarified that barriers clustered somewhat differently by context. Across settings, stigma, confidentiality concerns, financial burden, service complexity, and provider preparedness recurred repeatedly, but their relative salience differed. In South Korea, conditional reimbursement, uncertainty about where to obtain PrEP, and privacy concerns in healthcare settings appeared especially important [5,6]. Concerns about procedural knowledge, youth-unfriendly services, and dependence on limited community or pilot pathways were more visible in other Asian settings [14,15,17].

In Brazil and West African settings, misinformation, stigma, affordability, and uneven programmatic access were especially prominent [21,23,25,26]. Quantitative studies more often emphasized prevalence and correlates of awareness or uptake, whereas qualitative studies tended to examine disclosure anxiety, mistrust, stigma, and the social meaning of PrEP use.

Provider-related findings also recurred across multiple settings. Across the reviewed studies, PrEP uptake and continuation were influenced not only by user-level willingness but also by provider preparedness, confidentiality-sensitive communication, counseling quality, linkage support, and the clarity of follow-up procedures. In settings where access pathways were fragmented or poorly understood, the need for provider-side support in explaining eligibility, monitoring, and continuity of care appeared especially important. These patterns suggest that service delivery is shaped not only by structural access, but also by how consistently providers are able to communicate, coordinate, and support PrEP-related care. To reduce overlap across sections, the major barrier domains and their practice implications are consolidated in Table 4.

## 4. Discussion

Intersectionality also warrants more explicit attention. Although the Korean literature included in this review rarely reported detailed stratified analyses beyond broad participant characteristics, findings from other Asian settings suggest that younger MSM and other socially marginalized subgroups may encounter compounded barriers related to procedural knowledge, reliance on informal information channels, anticipated stigma, and lower confidence navigating services [14,15,16,17]. Future Korean research should therefore examine how age, student status, and other social locations shape PrEP engagement, while migration-related factors remain insufficiently examined in the currently available domestic literature.

This integrative review examined PrEP awareness, acceptance, and uptake among MSM populations and identified a persistent awareness-uptake gap shaped by interacting environmental, structural, and individual factors. The main implication of the review is that awareness alone is an inadequate endpoint for evaluating PrEP expansion. Instead, the more clinically and public-health-relevant question is whether awareness is supported by a service environment that allows potential users to move safely and predictably into care.

The clearest comparative contrast in the reviewed literature lies between the relatively institutionalized U.S. pathway and the more fragmented Korean pathway. However, the broader regional synthesis suggests that South Korea also shares important features with settings in which PrEP access depends on service visibility, culturally safe entry points, and sustained counseling rather than on financing alone. In Western Europe, financing and service routinization appeared comparatively stronger, yet uptake still depended on user literacy and clear access points [18,19]. In Brazil and West Africa, public or programmatic availability did not automatically overcome stigma, misinformation, or uneven service distribution [21,23,25].

Other Asian settings may be especially relevant for Korean interpretation. Thailand illustrates how community-linked and youth-affirming delivery models can improve engagement but still leave adherence and continuation vulnerable when follow-up systems are weak [14,15]. Chinese studies demonstrate that awareness can be widespread while uptake remains limited by uncertainty, self-efficacy, and practical navigation barriers [16,17]. Taken together, these findings suggest that South Korea’s challenges are neither entirely unique nor directly identical to the U.S. model; rather, they are better understood as part of a broader global pattern in which awareness, acceptability, and actual use diverge when delivery systems are fragmented or socially unsafe.

The findings also carry direct implications for nursing practice. Because nurses frequently occupy key positions in counseling, referral, testing coordination, medication education, and follow-up, they are well placed to reduce informational and relational barriers that weaken PrEP uptake. A nurse-centered PrEP navigation model may therefore be useful as a practical service strategy, particularly if it is combined with confidentiality-focused workflows, culturally affirming communication, and structured retention support. In addition, as long-acting injectable PrEP becomes more visible in the literature, nursing roles in regimen education, preference-sensitive counseling, and follow-up logistics may become increasingly important [32].

The review has several limitations. First, the included studies were heterogeneous in design, outcome measures, and analytic focus, which limited the possibility of quantitative synthesis and required interpretive thematic integration. Second, the use of broad database searches, including Google Scholar, increased retrieval breadth but also raised the possibility of uneven specificity during initial screening, although the Google Scholar search was restricted to the first 10 result pages for each major query combination. Third, although this revision broadens the comparative discussion beyond the United States and South Korea, the evidence base remained denser for some settings than for others, and the literature did not support equally detailed comparison across every global region. Fourth, although screening, appraisal, and data extraction were conducted independently by two reviewers, formal inter-reviewer agreement statistics were not calculated. Accordingly, the findings should be interpreted as an integrative comparative synthesis rather than as a uniform global estimate.

Despite these limitations, the study provides a useful framework for understanding why PrEP uptake may remain limited even when awareness is present. By framing the problem as an awareness–uptake gap rather than a simple deficit of knowledge, the review helps direct attention toward confidentiality, trust, institutional design, and the clinical work required to translate prevention knowledge into real-world preventive care.

## 5. Conclusions and Recommendations

This integrative review demonstrated that the gap between PrEP awareness and actual uptake among MSM populations cannot be adequately explained by lack of knowledge alone. Across South Korea and other global settings, the transition from awareness to actual use was shaped by the interaction of policy conditions, service accessibility, confidentiality, stigma, economic burden, and provider preparedness. The reviewed evidence consistently suggests that awareness becomes meaningful only when individuals are able to identify a clear entry point into care, trust that their privacy will be protected, and receive practical support for initiation and continuation. In this sense, the awareness–uptake gap should not be understood simply as a problem of insufficient information, but as a broader implementation problem that reflects how prevention services are organized, delivered, and experienced.

The comparative findings also indicate that the relative weight of these barriers differs across settings. In more institutionalized contexts, such as the United States and some European settings, formal policy support and reimbursement structures appear to reduce some economic and procedural obstacles, yet uptake may still remain limited when stigma, mistrust, complex engagement processes, or inadequate provider communication persist. In other Asian, Latin American, and African settings, the literature likewise shows that awareness or willingness alone does not guarantee uptake when access pathways are narrow, community linkage is weak, or service continuity remains uncertain. These comparisons suggest that South Korea is not facing an entirely unique problem, but rather a context-specific version of a broader global pattern in which prevention knowledge does not automatically translate into preventive behavior.

For South Korea, the findings of this review suggest that fragmented service pathways, uncertainty regarding where and how to obtain PrEP, concerns about privacy disclosure, and limited confidence in provider preparedness may represent particularly important barriers. Accordingly, efforts to improve PrEP uptake should move beyond awareness promotion alone. Policy and service strategies should focus on making PrEP access more visible, navigable, and trustworthy within routine healthcare and community health systems. This includes developing clearer referral and follow-up pathways, strengthening confidentiality-sensitive practices, reducing ambiguity surrounding service procedures, and providing communication that is culturally affirming and practically useful for potential users.

The findings also highlight meaningful implications for nursing practice. Nurses may be well positioned to reduce the awareness–uptake gap through counseling, service linkage, medication education, follow-up coordination, and continuity support. In particular, nurse-involved PrEP navigation may help address the gap between general awareness and actionable use by translating abstract knowledge into concrete steps for care engagement. However, such approaches should not be interpreted as universally established models; rather, they should be understood as evidence-informed and context-sensitive directions for service development. To support these roles effectively, provider training should include confidentiality-focused communication, nonjudgmental sexual health counseling, and practical guidance on initiation, monitoring, and long-term retention.

Future research should extend beyond measuring awareness, willingness, or acceptability in isolation. More implementation-oriented studies are needed to identify which service models most effectively support actual initiation, continuation, and retention in care. In the Korean context, future studies should also examine subgroup differences and practical service barriers in greater depth, including how age, social position, stigma experience, and familiarity with healthcare systems influence access to PrEP. In addition, service evaluation research is needed to determine whether nurse-supported counseling, community-linked referral systems, and structured follow-up models can improve uptake in real-world settings.

Taken together, this review supports a shift in emphasis from awareness promotion alone toward system-level, relationship-centered, and implementation-oriented PrEP strategies. Improving uptake among MSM populations will likely require not only broader knowledge dissemination, but also prevention systems that are confidential, accessible, culturally safe, and capable of sustaining engagement over time. In this regard, nursing and community-based practice may play an important role in transforming PrEP from an available intervention into a realistically usable form of HIV prevention.

## Figures and Tables

**Table 1:** Quality appraisal summary of included studies.

Appraisal Tool	Applicable Study Design	Studies Appraised, n	High-Quality Threshold/Summary Used in This Review
AXIS	Cross-sectional	120	≥15/20
Newcastle–Ottawa Scale	Cohort and case–control	16	≥7 stars
RoB 2.0	Randomized controlled trials	6	Low overall risk of bias
CASP	Qualitative	35	Positive judgment across the major domains
MMAT	Mixed-methods	10	≥4/5
Overall classification	All included studies	187	High quality: 139 (74.3%); Moderate quality: 48 (25.7%)

**Table 2:** General characteristics of included studies (N = 187).

Characteristics	Category	n (%)
Publication Year	2015–2019	67 (35.8)
	2020–2025	120 (64.2)
Study Region	North America	87 (46.5)
	Asia	35 (18.7)
	Europe/Others	65 (34.8)
Study Design	Quantitative	141 (75.4)
	Qualitative	35 (18.7)
	Mixed-methods	11 (5.9)
Population Composition	MSM-only samples	156 (83.4)
	Mixed key-population	31 (16.6)
Sample Size (N)	<100	31 (16.6)
	100–499	94 (50.3)
	500–999	38 (20.3)
	≥1000	24 (12.8)

**Table 3:** Comparative synthesis matrix for cross-regional analysis.

Region/Setting	Policy/Access Context and Key Barriers	Awareness/Acceptance Pattern	Implications for South Korea
South Korea	Conditional reimbursement and public support programs exist, but service pathways remain fragmented; key barriers include cost concerns, uncertainty about access, privacy concerns, stigma, and limited provider preparedness.	Awareness is improving, but procedural understanding and sustained uptake remain limited.	Need clearer navigation, confidentiality protections, and structured follow-up.
United States	Policy recommendations, insurance coverage, and clinical guidelines are more institutionally aligned, but uptake remains constrained by stigma, complex care engagement, provider bias, and retention challenges.	Awareness is comparatively high, but uptake is still limited by stigma and trust barriers.	Illustrates the value of aligning policy, financing, and service protocols.
Other Asian settings	Community-led or pilot-site delivery supports access in settings such as Thailand, but implementation remains uneven; major barriers include procedural knowledge gaps, youth-unfriendly services, stigma, side-effect concerns, and reliance on limited delivery sites.	Awareness is often moderate to high, yet major drop-off persists from awareness to willingness, uptake, or adherence.	Suggests that financing alone is insufficient without community-linked delivery, affirming counseling, and clear service navigation.
Europe/other high-income settings	PrEP is more often integrated into reimbursed or publicly supported preventive services with identifiable hospital or community entry points, but uptake still varies according to knowledge of dosing options, uneven geographic access, lower self-efficacy, and limited provider or community discussion.	Awareness is generally high, but uptake still varies according to regimen literacy, perceived need, and visibility of access points.	Shows that even when financing improves, uptake still depends on literacy about PrEP modalities and accessible counseling pathways.
Latin America (including Brazil, if eligible)	Public-sector expansion broadens formal access in Brazil, but services remain concentrated and unevenly distributed; internalized homonegativity, misinformation, stigma linking PrEP to hidden HIV status, racial inequity, and service concentration continue to limit use.	Awareness and willingness are present, yet consistent current use remains lower than expected among eligible MSM.	Highlights how stigma and concentrated service delivery can limit uptake even when formal access exists.
Sub-Saharan Africa (if eligible, MSM-focused studies were included)	Community-based and demonstration settings generate interest, but routine access remains uneven and often donor-dependent; key barriers include low awareness, stigma, fear of side effects, affordability concerns, and uncertainty about sustained access and adherence.	Willingness frequently exceeds baseline awareness; once informed, many MSM express strong interest or acceptability.	Reinforces the importance of culturally safe communication and reliable delivery systems when awareness does not automatically lead to use.

**Table 4:** Consolidated barrier domains and practice implications.

Barrier Domain	Typical Manifestations	How the Gap Widens	Implications for Nursing and Service Design
Social and psychological	PrEP stigma, anticipated judgment, disclosure anxiety, moralization of PrEP use	Awareness does not become willingness when anticipated judgment outweighs perceived preventive benefit.	Use affirming counseling, normalize PrEP as prevention, and protect privacy in communication and documentation.
Structural and system	Unclear entry points, fragmented monitoring, conditional reimbursement or cost burden, travel and time burden	Interested users delay or abandon initiation because the pathway into care is complex, costly, or hard to trust.	Provide clear referral pathways, step-by-step navigation, appointment coordination, and confidentiality safeguards.
Informational and relational	Surface awareness without procedural knowledge, uncertainty about side effects, monitoring, and regimen options	Users may know PrEP exists but still feel unprepared to start or sustain it.	Provide regimen education, written follow-up guidance, and decision support tailored to practical concerns.
Provider-related	Limited provider familiarity, inconsistent counseling, weak linkage and retention support	Care transitions break down after eligibility assessment or early initiation.	Strengthen nurse and provider training, standardize counseling and follow-up, and build retention support systems.

## Data Availability

No new datasets were generated or analyzed in this study. Data sharing is not applicable to this article.

## Public Involvement Statement

No patients or members of the public were involved in the design, conduct, reporting, or dissemination plans of this review.

## Guidelines and Standards Statement

This integrative review was conducted according to the five-step methodology described by Whittemore and Knafl and was reported with reference to PRISMA guidance.
